# Insights into Structure and Function of Growth Arrest Specific 2 (GAS2)

**DOI:** 10.7150/jca.102893

**Published:** 2025-01-01

**Authors:** Wenjuan Ma, Jiawei Lin, Rongyao Ma, Yang Bai, Di Wu, Xiuyan Zhang, Haixia Zhou, Yun Zhao, Lei Zhang

**Affiliations:** 1 Cyrus Tang Medical Institute, Collaborative Innovation Center of Hematology, Soochow University, Suzhou, China.; 2 The First Affiliated Hospital of Soochow University, Jiangsu Institute of Hematology, NHC Key Laboratory of Thrombosis and Hemostasis, Suzhou, China.; 3 National Clinical Research Center for Hematologic Diseases, Suzhou, China.; 4 MOE Engineering Center of Hematological Disease, Soochow University, Suzhou, China.

**Keywords:** GAS2, Calpain-2, Apoptosis, Tumor suppressor gene, Oncogene

## Abstract

Growth arrest specific 2 (GAS2) is a microfilament-associated protein, which is widely distributed in human tissues. It exerts a pivotal influence on various cellular processes, including cytoskeletal regulation, cell cycle progression, apoptosis, and senescence. GAS2 has a dual function in cancer cell growth: on the one hand, it enhances the sensitivity of cancer cells to chemoradiotherapy and prevents malignant transformation of normal cells; but on the other hand, it maintains the growth of cancer cells. GAS2 regulates the cellular activity of Calpain-2, a calcium-dependent protease, by acting as an endogenous inhibitor of the enzyme. The N-terminus of GAS2 binds to Calpain-2, while its C-terminus acts as an inhibitor of the protease activity of Calpain-2. The functional outcome of GAS2 is highly dependent on the specific substrates of Calpain-2 and cellular environment, particularly within tumor cells. Despite garnering increasing attention and a growing body of related research, a systematic review of GAS2 remains absent. This review aims to elucidate the structural and functional aspects of GAS2, with a particular emphasis on its implications in cancer. By comprehensively detailing its role and research progress in malignancies, this review endeavors to furnish novel insights for enhancing the therapeutic strategies against diseases, particularly cancers.

## Introduction

Growth arrest specific (GAS) superfamilies include the GAS1 family, GAS2 family…GAS8 family, and there are four members of the GAS2 family: GAS2, GAS2L1 (GAS2-like 1), GAS2L2 (GAS2-like 2) and GAS2L3 (GAS2-like 3). Although GAS superfamilies are defined as a group of growth arrest specific proteins, different members differ significantly in terms of expression patterns, and biological functions 1-3. In 1988, Schneider et al. first identified a cluster of genes up-regulated in murine fibroblasts NIH3T3 cells in response to serum starvation and density-dependent inhibition, among which is named as *Gas2* 4. During growth arrest in NIH3T3 cells, Gas2 protein localizes to the actin fibers near the cell membrane border and along stress fibers 5. Subsequent studies have found that the distribution of human GAS2 protein mirrors that observed in murine cells 6. GAS2 plays a pivotal role in regulating a spectrum of fundamental cellular processes, encompassing cytoskeletal regulation, cell cycle progression, apoptosis, and senescence 5, 7-9. Particularly in apoptosis, GAS2 exhibits a dual role: as an effector of the apoptotic process, which is directly regulated by caspase enzymes 8, 10, 11, or as a regulator of the apoptotic process, which modulates the susceptibility of specific signals to the apoptotic response 12, 13. This complexity extends to its signaling pathways in cancer cells, where both tumor-suppressive and tumor-promoting activities have been observed 9, 14. In addition, the roles of GAS2 in cancer development, progression, metastasis and relapse might be not the same. Consequently, targeting GAS2 signaling for cancer therapy remains in the preclinical stage, necessitating a detailed understanding of its roles across different cancer types. Despite available publications on the structure and function of GAS2, a systematic review of this topic is lacking. In this review, we aim to summarize recent research on the detailed structural characteristics and biological functions of GAS2 signaling, with a particular focus on its roles in different types of cancers.

## Structure and Expression

### Structural features of GAS2

**Figure 1:**
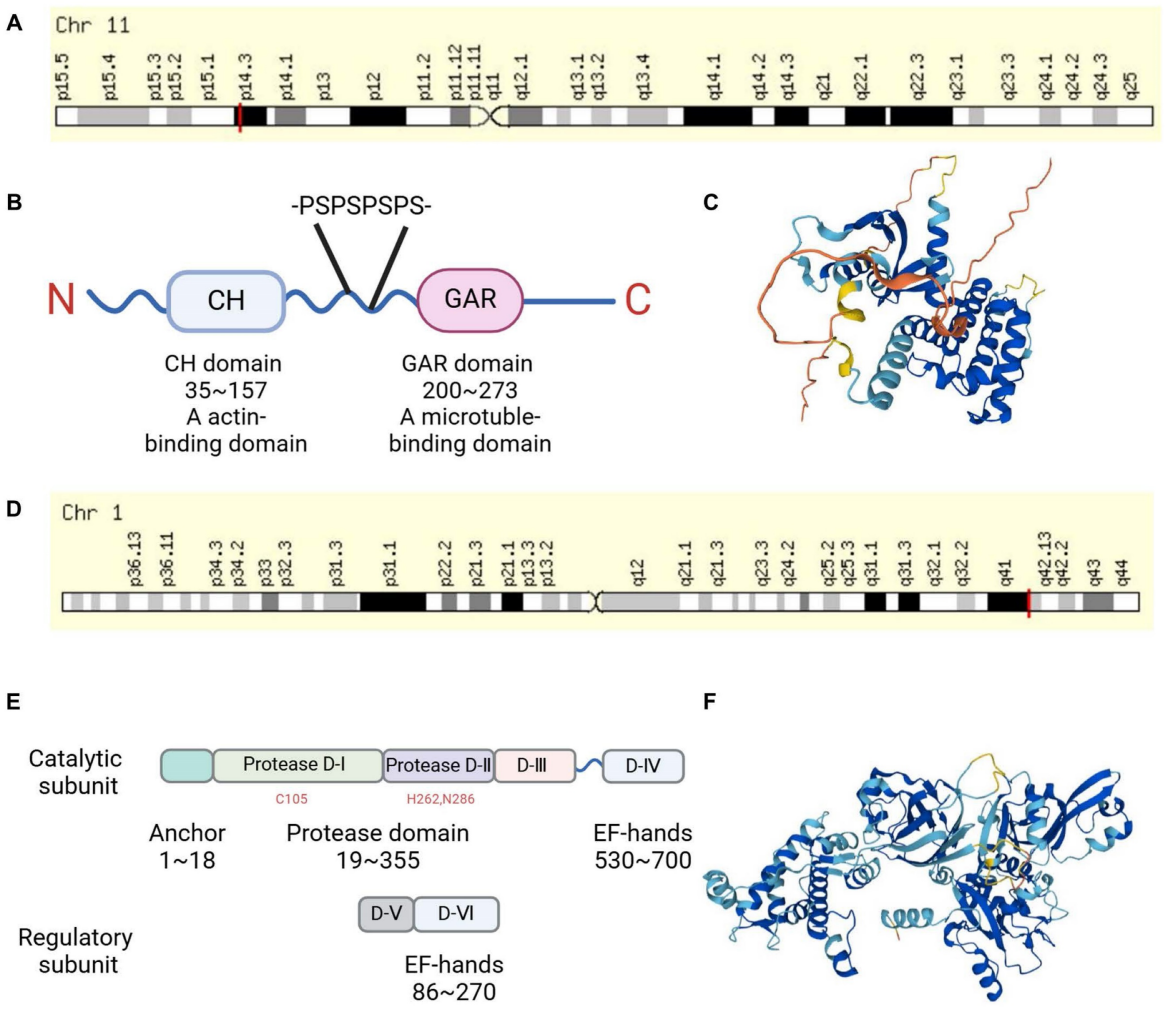
** The chromosomal localization and structure of GAS2 and Calpain-2.** (A) The GAS2 encoding gene is located on human chromosome 11p14.3. (B) GAS2 contains a CH domain and a GAR domain, there is a flexible region with four proline-serine (P-S) repeats between them. (C) 3D protein structure map of GAS2 from the UniProt database (https://www.uniprot.org/). (D) The Calpain-2 encoding gene is located on human chromosome 1q41. (E) Calpain-2 consists of a catalytic subunit and a regulatory subunit. (F) 3D protein structure map of Calpain-2 from the UniProt database. CH, calponin-homology; GAR, growth arrest-specific related.

Gas2 was originally identified in murine fibroblasts under growth arrest conditions 4. It is localized on murine chromosome 7 and shares homology with the *GAS2* gene located on human chromosome 11p14.3-p15.2 6 **(Figure 1A)**. The mRNA of mouse and human *GAS2* encodes a product of 314 and 313 amino acids, respectively, with a molecular weight of approximately 36 kDa. It was found that *GAS2* mRNA is expressed in most human tissues through RT-PCR, while GAS2 protein is highly expressed in the liver, lungs, and kidneys as observed through immunoblot analysis, 6 consistent with findings in murine tissues 4. A study comparing GAS2 protein sequences from various species, including human, dog, mouse, cow, and *Xenopus laevis* by bioinformatics, revealed a high degree of evolutionary conservation, suggesting that GAS2 has similar biological functions in different species 7. The GAS2 protein structure comprises the N-terminal CH domain (calponin homology domain), with amino acid sequences in human ranging from 35 to 157, and the C-terminal GAR domain spanning amino acids 200 to 273. There are two low-complexity regions between the CH and GAR domains, the second of which contains four proline-serine (P-S) repeats, making the structure of this region more flexible 7 **(Figure 1B-C)**.

### GAS2 is an endogenous inhibitor of Calpain-2

**Figure 2:**
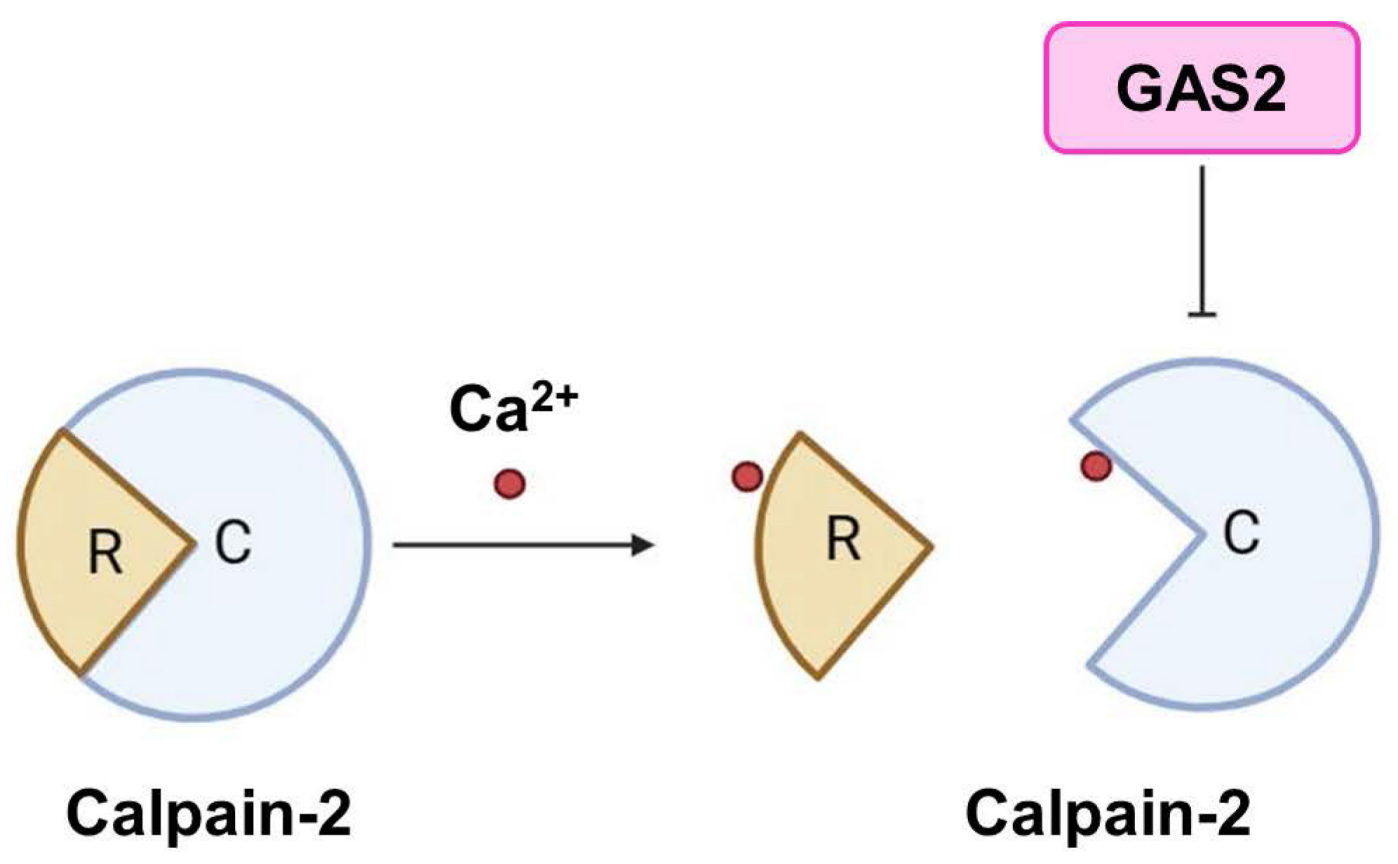
** GAS2 is an endogenous inhibitor of Calpain-2.** GAS2 has the ability to inhibit the activation of Calpain-2 by binding calcium ions. R, regulatory subunit; C, catalytic subunit.

Calpains are a family of calcium ion-activated proteases belonging to the cysteine protein hydrolase superfamily. They can be categorized into two main groups: tissue-specific and non-tissue-specific proteases, based on their distributional characteristics 15. Calpain-1 and Calpain-2 are non-tissue-specific isozymes. Calpain-2 can be activated by millimolar levels of calcium ions, and is also known as m-Calpain. It exists as a heterodimer comprising an 80 kD catalytic subunit and a 30 kD regulatory subunit **(Figure 1D-F)**. Structurally, the catalytic subunit consists of four domains (I, II, III, and IV), while the regulatory subunit consists of two domains (V and VI). Both the IV and VI domains contain four "EF fingers" structures, which bind calcium ions and form heterodimers with each other. Calcium binding to these EF fingers induces conformational changes in the IV and VI domains, leading to intermolecular autolysis, as well as dissociation of the catalytic and regulatory subunits, ultimately resulting in Calpain-2 activation 16. A study has revealed a link between Calpain-2 and human T-cell leukemia, showing decreased expression of *Calpain-2* in this context. Furthermore, overexpression of the active state of Calpain-2 has been demonstrated to inhibit the growth of leukemia cells 17. Interestingly, Berretti et al. found that Gas2 binds to the C-terminus of the large subunit of m-calpain by a yeast two-hybrid screen. *In vitro* experiments showed that the binding of Gas2 to m-calpain inhibits its protease activity, thus Gas2 is an endogenous inhibitor of m-calpain 12 **(Figure 2)**. Another study proposed that the N-terminus of GAS2 and GAS2DN (the dominant negative form of GAS2) bind to the III and IV subunits of m-Calpain, with the C-terminus of GAS2 primarily exerting inhibitory effects 12. Furthermore, Calpastatin is a recognized endogenous Calpain inhibitor. Its inhibitory structural domain comprises three highly conserved regions (A, B, and C), with the B region predominantly responsible for inhibitory functions, while the A and C regions can bind to m-calpain and are dependent on free Ca^2+^ for binding, sharing the same binding domain as Gas2 18, 19.

### Normal and abnormal expression of GAS2

**Figure 3:**
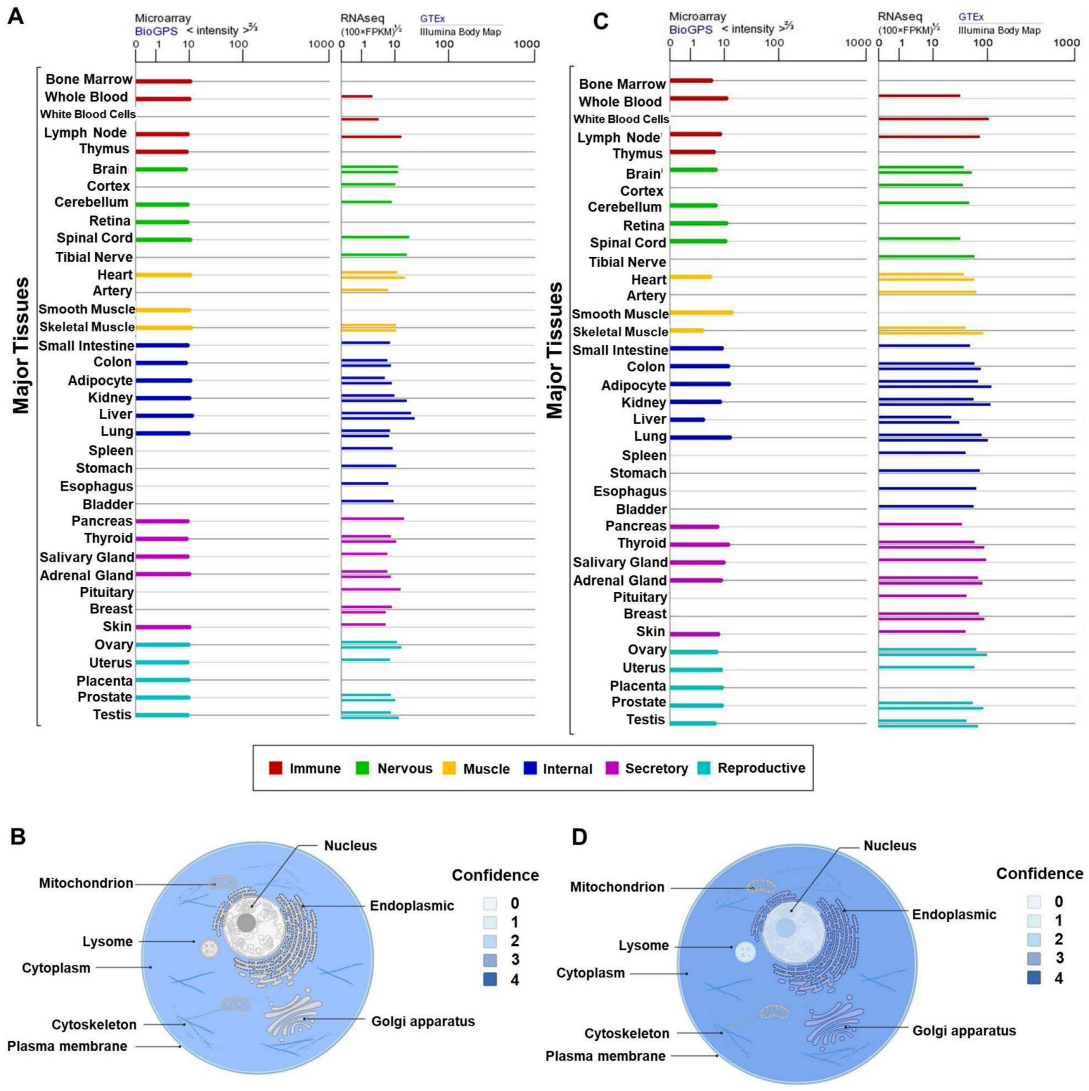
** Analysis of GAS2 and Calpain-2 expression.** (A) The expression levels of GAS2 in various tissues and different blood cells. (B) The subcellular localization of GAS2. (C) The expression levels of Calpain-2 in various tissues and different blood cells. (D) The subcellular localization of Calpain-2. The data are sourced from the Genelibs database (https://www.genelibs.com).

Lee et al. found that *Gas2* was expressed in mouse interdigital tissues, chondrogenic and myogenic regions. At embryonic developmental stages of day 10.5, 11.5, and 13.5, western blotting revealed a rapid upregulation of *Gas2* expression. Immunohistochemical analysis demonstrated high Gas2 expression in soft connective tissues of the head and trunk, and intervertebral tissues, while TUNEL analysis indicated a notable presence of cell death in these regions. Additionally, a small amount of *Gas2* expression was detected in developing lung, renal cortex, lens of the eye, and vertebral cartilage located cranially. In the early stages of chondrogenesis, *Gas2* expression was absent in the limb mesenchymal cells, as they aggregated to form chondrogenic nodules. However, as these nodules progressed in development, *Gas2* was subsequently expressed in the periphery of these nodules and the surface cells of developing cartilage in the limb. Interestingly, at more advance stages of development, *Gas2* expression was observed in cells located at the center of cartilage tissue, suggesting a role in normal cartilage development 10. Furthermore, an overview of *GAS2* and *Calpain-2* expression patterns in various normal human tissues or blood cells was provided. As depicted in **Figures 3A** and **3C**, *GAS2* and *Calpain-2* exhibit comparable distribution characteristics, with high expression levels observed in multiple tissues, including the liver, pancreas, and lung, among others.

Serum-starved NIH 3T3 cells were subjected to various cytokinesis stimuli, including 20% serum, platelet-derived growth factor (PDGF), lysophosphatidic acid (LPA), and phorbol ester (PMA). Interestingly, all these stimuli led to the down-regulation of Gas2 protein expression. Notably, the addition of 20% serum, PDGF, and PMA induced hyperphosphorylation of the Gas2 protein, accompanied by membrane ruffling at the Gas2 localization. Conversely, LPA, a specific inducer of stress fiber formation, failed to trigger Gas2 hyperphosphorylation 5, 20. The mRNA level of *Gas2* in mouse F9 embryonal carcinoma cells remained relatively stable following treatment with all-trans retinoic acid. However, upon nutrient deprivation during differentiation, the mRNA levels of growth arrest-related genes reverted to those characteristic of the stem cell stage. Notably, the mRNA levels of Gas2 and other genes increased rapidly after retinoic acid treatment in the nutrient-deprived medium, indicating that *Gas2* expression in mouse F9 embryonic carcinoma cells is influenced by varying nutritional conditions 21.

An exploration into breast carcinogenesis using oocyte-to-tumor cell reprogramming revealed the capacity of oocyte extracts to induce the expression of tumor suppressor genes. Notably, the expression of *GAS2* and other genes was low in breast cancer MCF-7 and HCC1954 cells. However, extracts from Salamander and *Xenopus* oocytes were able to induce the re-expression of *GAS2* and other genes, as did extracts from mouse embryonic stem cells. Analysis of histone modifications in the promoters of reactivated genes revealed elevated levels of histone H3 lysine 4 trimethylation (H3K4me3), a modification associated with transcriptional activity, particularly evident on promoters such as *GAS2* 22. *GAS2* mRNA was detected via *in situ* hybridization in paraffin sections, revealing higher expression levels in liver cancer tissues compared to adjacent liver tissues. Positive expression of *GAS2* was correlated with apoptosis, as indicated by TUNEL and immunohistochemical assays, while showing no significant correlation with cell proliferation. These findings suggest a potential role for GAS2 in primary hepatocellular carcinoma by influencing cell apoptosis 23.

Myeloproliferative disorders encompass idiopathic myelofibrosis (IM), polycythemia vera (PV), essential thrombocytosis (ET), and chronic myeloid leukemia (CML). The JAK2V617F mutation is detectable in individuals with IM, PV and ET, where constitutively activated JAK2 partially accounts for the abnormal proliferation of myeloid cells and their heightened sensitivity to cytokines. However, JAK2V617F may not serve as the pathogenic initiating factor 24-26. In a study by Vannucchi et al., a comparison of CD34^+^ cells from the bone marrow of IM patients with those of normal donors revealed a high expression of GAS2. Subsequent RT-qPCR analysis performed on CD34^+^ cells from bone marrow samples of patients with IM, PV, ET, as well as normal donors, confirmed significantly elevated levels of GAS2 expression across all three diseases 27.

In addition, we have summarized the protein subcellular localization of GAS2 and Calpain-2 based on COMPARTMENTS localization data, which offers unified and visualized evidence of protein subcellular localization (**Figure 3B**, **3D**). Notably, GAS2 demonstrates relatively high confidence scores for localization in the cytoskeleton and cytoplasm. Furthermore, our analysis reveals high confidence in the subcellular localization of Calpain-2 in the plasma membrane, cytoplasm, lysosome, vacuole, endoplasmic reticulum, Golgi apparatus, cytoskeleton and nucleus.

## Biological Function

### Cytoskeleton

The cytoskeleton is a dynamic network of protein fibers within the cytoplasm of cells, playing crucial roles in cell morphology, movement, division, and other cellular processes. It comprises three main types of protein fibers: microtubules, microfilaments, and intermediate filaments. Microtubules consist primarily of tubulin, while microfilaments are composed of actin 28. Schneider et al. demonstrated that Gas2 is a component of the microfilament system, although its direct interaction with actin has yet to be conclusively determined 5. Subsequent experiments revealed that overexpression of C-terminally truncated forms of Gas2∆276-314 and ∆236-314 induced significant alterations in the actin cytoskeleton and cellular morphology, primarily affecting the microfilament system without influencing intermediate filaments and microtubules. Transfection of the Gas2 C-terminus in COS-7 fibroblasts showed co-localization with the endogenous microtubule system through immunofluorescence analysis 26. Further investigations using cryo-confocal microscopy demonstrated that both full-length Gas2 and its GAR domain co-localized with the microtubule system in the cortex of *Xenopus* embryonic cells. Cytoskeleton co-sedimentation assays revealed Gas2 co-precipitated with microtubules, while electron microscopy revealed the formation of highly ordered structures by Gas2 and microtubules. Microscopic injection of either full-length Gas2 or its GAR domain inhibited cell division in *Xenopus* embryonic cells, suggesting that Gas2 inhibits cell division by binding to microtubules via its C-terminal GAR domain 7. We hypothesize that the N-terminus of GAS2 binds to actin, while its C-terminus binds to microtubules (**Figure 1B**, **Table 1**).

### Cell cycle

The cell cycle, a fundamental process in which a cell divides to generate two daughter cells, consists of five phases in eukaryotes: G0, G1, S, G2, and M 29. The GAS2 protein was initially identified in mammalian cells, where it was found to play a role in cell cycle regulation 4, 5. Subsequent studies have investigated the specific relationship between GAS2 and the cell cycle in various cell types. For instance, Gas2 protein levels were found to increase upon serum starvation in the murine NIH 3T3 cell line 4, 30, and to a lesser extent in embryonal carcinoma F9 cells 21, but not in the murine keratinocyte MSCP5 cells, 31 suggesting the cell specificity of Gas2 function. Despite Gas2 protein expression being up-regulated during cell growth inhibition, its levels remained relatively stable during the transition from G0 to G1 phase due to its long half-life. During this phase transition, Gas2 was observed to undergo phosphorylation. Subsequent studies revealed that upon 5 minutes of serum stimulation in growth-inhibited NIH 3T3 cells, Gas2 underwent phosphorylation at serine sites, maintaining phosphorylation levels for up to 3 hours. Furthermore, Gas2 hyperphosphorylation was associated with the formation of membrane ruffling 5, 20. Furthermore, Gas2 expression was found to vary in mouse primary hepatocytes with different ploidy levels 32. Overexpression of Gas2 in *Xenopus* embryonic cells inhibited cell division and led to the formation of multinucleated cells 7. Similarly, upregulation of GAS2 in the hepatocellular carcinoma SK-Hep1 cells suppressed the G1-to-S transition of the cell cycle 23, 30. In contrast, GAS2 expression in two T-cell acute lymphoblastic leukemia cell lines (Jurkat and CCRF-CEM) promoted the G1-to-S transition of the cell cycle 33. These findings underscore the diverse roles of GAS2 in cell cycle regulation, with its effects varying depending on cell type and context (**Table 1**).

### GAS2 plays a dual role in apoptosis

Apoptosis, a form of programmed cell death, can be initiated by both internal and external signals. It is characterized by distinctive morphological changes and the activation of specific caspase (cysteine-dependent aspartate specific protease) family and mitochondrial control pathways 34, 35. In NIH 3T3 cells, overexpression of Gas2 or Gas2∆295-314 did not reveal significant cellular morphology changes. However, overexpression of Gas2 variants with C-terminus excision (∆276-314 and ∆236-314) induced substantial alterations in the actin cytoskeleton and cellular morphology, which were similar to those occurring in apoptosis. This suggests that these variants may not interfere with endogenous Gas2 function, but rather but rather could confer a gain of function 11. When subjected to serum starvation, BALB/c LT-2809 cells demonstrated a notable induction of apoptosis. Concurrently, there was a significant up-regulation in Gas2 expression, accompanied by its hydrolyzation. These findings imply a plausible mechanism through which Gas2 may heighten the susceptibility of cells to apoptosis 13. In a separate study, human osteosarcoma U2OS cells and MEFs (mouse embryonic fibroblasts) were exposed to various DNA-damaging agents including ultraviolet irradiation, etoposide, and methyl mesylate. It was observed that overexpression of Gas2 effectively heightened the susceptibility of cells to apoptosis. This effect was mediated by Gas2's interaction with m-calpain, leading to the inhibition of m-calpain-mediated degradation of p53 and consequently enhancing the stability and transcriptional activity of the p53 protein. Conversely, the dominant-negative form of Gas2, Gas2∆171-314, bound to m-calpain without inhibiting its activity, thereby nullifying Gas2's effect on p53 12.

Various apoptotic stimuli, such as serum starvation and exposure to DNA-damaging agents, have been shown to induce apoptosis in NIH 3T3 cells. *In vitro* mutagenesis has unequivocally demonstrated the essential role of the aspartic acid residue at position 279 in the proteolytic cleavage of Gas2, implicating the potential involvement of an interleukin-1 beta-converting enzyme-like protease in the apoptotic processing of Gas2 11. In a separate investigation, apoptosis induction in human breast cancer MCF-7 cells via UV treatment revealed that caspase-3, and caspase-7, recognize and cleave aspartic acid at position 279 of GAS2 during the early stages of apoptosis. This cleavage of GAS2's C-terminal region by caspase-3 has been linked to subsequent binding to F-actin and a specific reorganization of the microfilament system, indicating its significance in inducing morphological changes in apoptotic cells 8. Furthermore, Gas2 has been implicated in the apoptotic process of mouse interdigital tissues, where its C-terminal cleavage and caspase-3 precursor activation have been observed. These findings suggest a potential collaborative role of Gas2 and caspase-3 in the apoptotic events within interdigital tissues during mouse embryonic development 10.

The above studies suggest that GAS2 may play a dual role in apoptosis, on the one hand, GAS2 acts as a regulator of apoptosis, and regulates the susceptibility of cells to special signals of apoptosis; and on the other hand, as an effector, which is cleaved by caspases during apoptosis and participates in the morphological changes of cells (**Table 1**).

### Cancer

**Figure 4:**
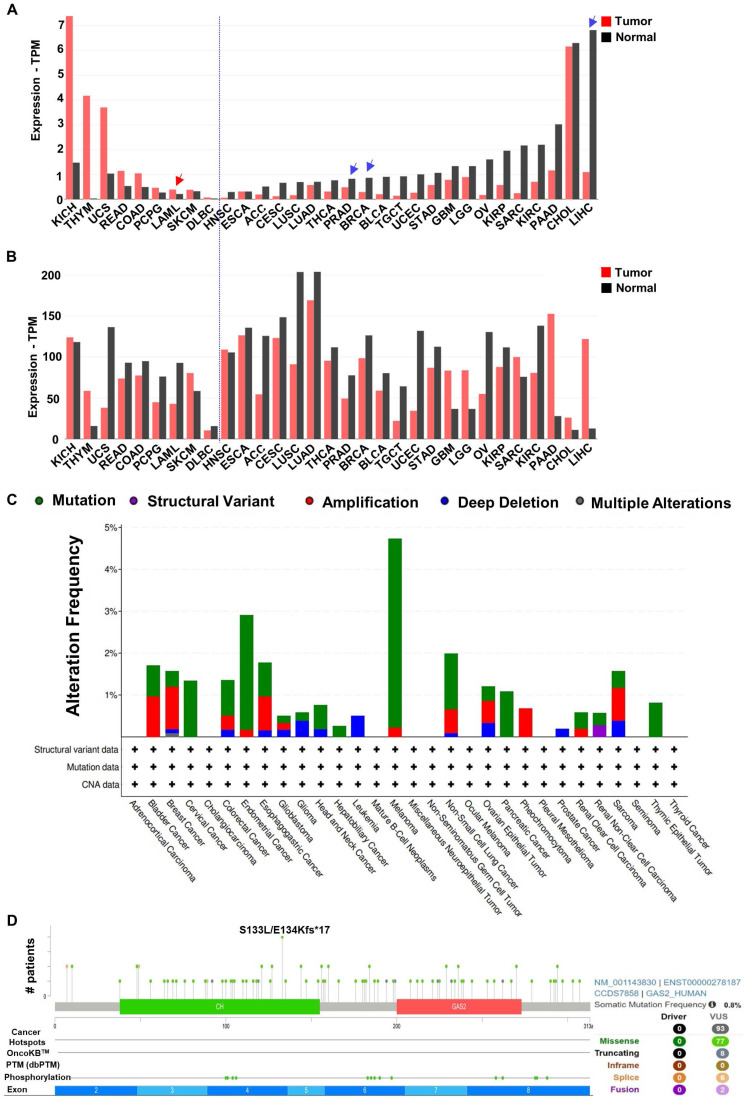
** Expression and Mutation analysis of GAS2 and Calpain-2 in TCGA tumors.** (A) The expression of GAS2 in different tumors and healthy tissues. The demonstrated cancer-promoting (red arrowhead) or anticancer (blue arrowhead) effects of GAS2 have been labeled accordingly. (B) The expression of Calpain-2 in different tumors and healthy tissues. The data is extracted from the database GEPIA2 (http://gepia2.cancer-pku.cn). (C) The alteration frequency of different mutations of GAS2 in various cancers. (D) Schematic representation of GAS2 mutation types and specific mutation sites. The data is sourced from the cBioPortal database (https://www.cbioportal.org/). Cancer abbreviation and corresponding full name listed in **Table S1**.

Analysis of the GEPIA2 database revealed distinct expression patterns of GAS2 and Calpain-2 among various cancer patients (**Figure 4A**, **4B**). In general, compared with normal cells, if GAS2 is highly expressed in the tumor cells, it has a cancer-promoting effect, and if GAS2 is low expressed in the tumor cells, it has a cancer-inhibiting effect. Additionally, examination of TCGA database data allowed us to characterize the mutation landscape of GAS2 across different cancer types, where mutation rates were generally below 5% (**Figure 4C**). Furthermore, we investigated specific mutation frequencies within GAS2 across all cancer cases. Notably, **Figure 4D** illustrates that the most prevalent missense mutation, S133L/E134Kfs*17, was observed in only four cancer cases. These findings underscore the tissue-specific roles of GAS2 in cancer pathogenesis (**Table 2**). While mutations in GAS2 are infrequently reported in human cancers, elevated GAS2 expression has been documented in leukemia, colorectal cancer, among others 36-38. In these malignancies, increased GAS2 activity correlates positively with disease progression, indicating its potential role as an oncoprotein. Conversely, GAS2 is downregulated in breast cancer, hepatocellular carcinoma, and prostate adenocarcinoma, where it may function as a tumor suppressor 22. Thus, a comprehensive understanding of GAS2's role in different cancer types is crucial for developing targeted antineoplastic therapies.

#### Tumor promoting activity

**Figure 5:**
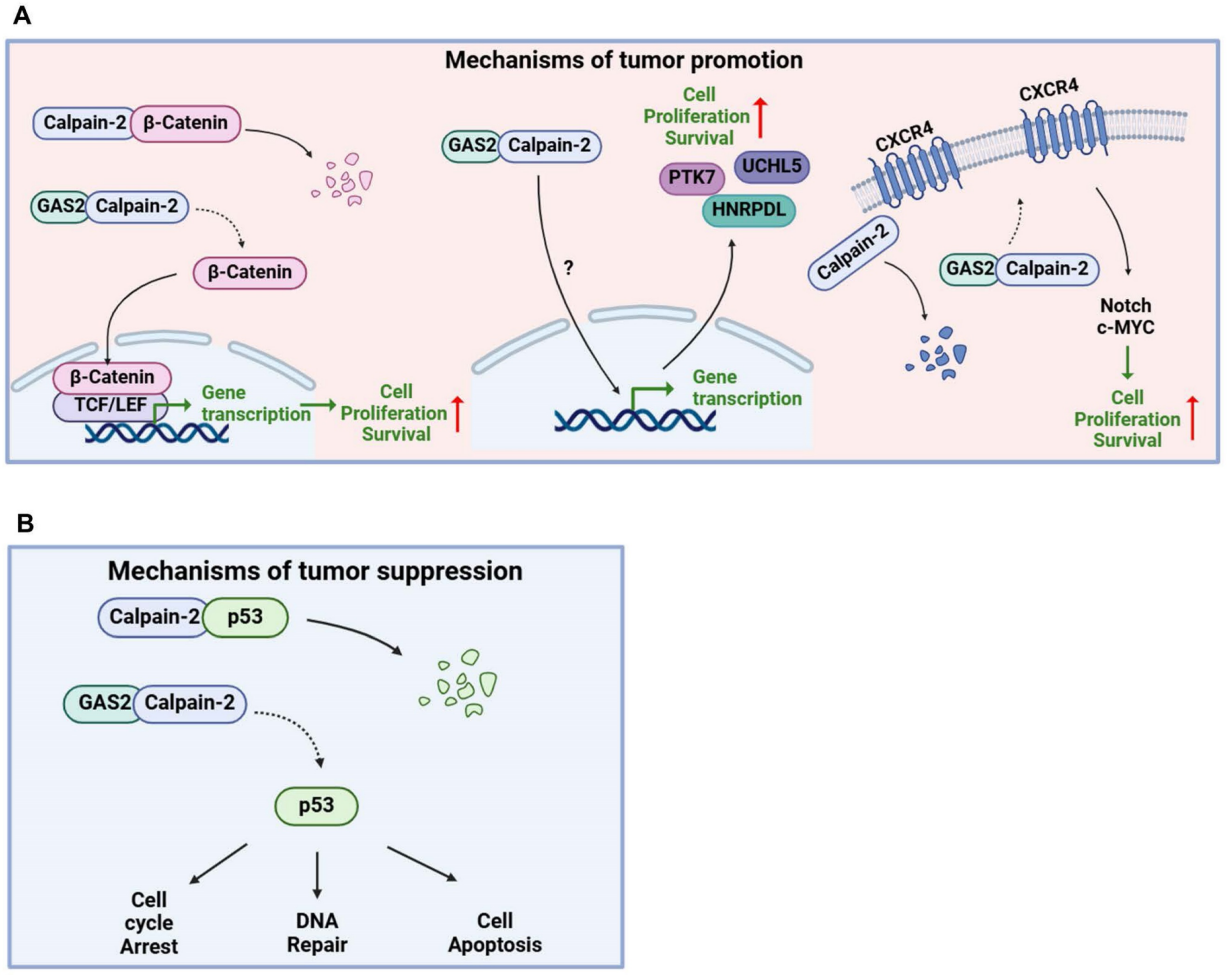
** Opposite roles of GAS2 in cancers.** (A) Tumor promoting activity of GAS2: GAS2 inhibits the activity of Calpain-2, resulting in the accumulation of various Calpain-2 substrates, including β-Catenin, CXCR4, and others, which ultimately promotes cancer progression. Additionally, the interaction between GAS2 and Calpain-2 enhances the expression of downstream genes via unidentified pathways, further facilitating cancer progression. (B) Tumor suppressive role of GAS2: GAS2 inhibits the activity of Calpain-2, resulting in the accumulation of p53, which ultimately inhibits cancer progression.

GAS2 promotes tumor progression through different mechanisms in different cancers (**Figure 5A**). Independent of the GSK3β/proteasome pathway, the GAS2-Calpain2 axis functions as an alternative mechanism for inducing β-catenin degradation. Targeting *GAS2* using RNA interference or overexpression of *GAS2DN* has demonstrated efficacy in inhibiting the proliferation of colon cancer cells (HCT116) *in vitro* 39. Scholars in Taiwan have shown that *GAS2* overexpression promotes the growth of colon cancer cells (SW480) growth *in vitro* 36. In addition, Wu et al. reported a significant positive correlation between the upregulation of GAS2 expression and the stage of colorectal cancer 37. The interferon consensus sequence binding protein (Icsbp/Irf8) has been identified as a regulator of Calpain-2 activation by suppressing *GAS2* expression. This suppression leads to the degradation of β-catenin and the termination of emergency granulopoiesis. Conversely, in bone marrow progenitors with BCR-ABL^+^ (a fusion gene associated with chronic myeloid leukemia) or Icsbp^-/-^, high GAS2 expression stabilizes β-catenin, thereby promoting human leukemogenesis 40. In gene expression profiling studies conducted with samples from CML patients, GAS2 was identified as highly expressed, with a notable upregulation observed during the transition from the chronic phase to the acute phase of the disease 41-43. The Eklund team discovered that the GAS2/Calpain-2 axis induces β-catenin accumulation and enhances cell growth in BCR-ABL^+^ cells. This axis leads to the accumulation of STAT5 and Xiap1, while the accumulated β-catenin activates Survivin, rendering BCR-ABL^+^ cells resistant to apoptosis 44-48. In our laboratory studies, we observed a significant upregulation of GAS2 expression in CD34^+^ cells from CML patients compared to normal control cells. Targeting GAS2 not only significantly inhibited CML cell proliferation but also enhanced the sensitivity of these cells to Imatinib, a commonly used therapeutic agent in CML treatment. Additionally, GASDN exhibited relatively specific inhibition of colony formation in CML progenitor cells while exerting minimal effects on normal hematopoietic progenitor cells. Treatment with GAS2DN resulted in significant downregulation of several genes, including HNRPDL, PTK7, and UCHL5, suggesting a potential mechanism whereby GAS2DN regulates downstream gene expression, thereby exerting its anti-cancer effects. 49. Furthermore, our research has demonstrated that HNRPDL plays a role in malignant transformation within hematopoietic cells, promoting the growth of CML cells and influencing their response to drugs, partially through its regulation of PBX1 50. In addition, GAS2 exhibits abnormal expression in other types of leukemia, including acute lymphoblastic leukemia (T-ALL), where it regulates T-ALL through pathways involving CXCR4 and c-MYC 14, 38. These findings collectively suggest that GAS2 holds promise as a novel biomarker and potential therapeutic target across multiple cancer types.

#### Tumor repressive activity

GAS2 is recognized as a tumor suppressor gene, primarily exerting its function through the stabilization of p53 (**Figure 5B**). A research by Schneider's group revealed that GAS2 enhances the sensitivity of cancer cells to etoposide and methanesulfonic acid by promoting p53 protein stability 12. eIF4E plays a crucial role in initiating mRNA translation by binding to their 5'-caps. This activity is typically inhibited by eIF4E-binding proteins (4E-BPs). In MEFs lacking 4E-BPs, eIF4E leads to an increase in Gas2 protein expression. This heightened Gas2 level subsequently stabilizes p53 by inhibiting m-calpain activity. As a result, there is an increase in p53-dependent cellular senescence and suppression of Ras-induced malignant transformation. Conversely, silencing Gas2 expression through RNA interference reduces the number of senescent cells and promotes cell proliferation 51. Furthermore, studies utilizing *Xenopus* embryonic cells have shown that both Gas2 and N-terminal deletion variants of Gas2 inhibit cell growth, suggesting a role in cell division inhibition through C-terminal binding to microtubule proteins 52. GAS2 expression does not directly induce cell apoptosis but enhances etoposide-induced apoptosis in human breast cancer MCF7 cells, which express wild-type p53 53. Moreover, Zhu et al. demonstrated that the overexpression of the mutant form of the hepatitis B virus encoded X antigen (HBx) in hepatocellular carcinoma binds directly to the GAS2 promoter, attenuating GAS2 expression and promoting hepatocyte proliferation and tumorigenicity 30. Collectively, these findings underscore the multifaceted role of GAS2 in tumor suppression, either by inhibiting tumor cell proliferation or sensitizing tumor cells to chemotherapy drugs.

## Prospective

The growth arrest specific protein GAS2, known for its involvement in regulating various cellular functions such as the cell cycle, apoptosis, and cancer, operates through mechanisms including protein degradation pathways 1, 14. Protein degradation is an important way to regulate protein expression. Abnormal protein degradation pathways can be involved in a variety of human diseases 54. Specifically, Calpain-2, a crucial protease in protein degradation, is inhibited by GAS2. Aberrant expression of GAS2 leads to decreased Calpain-2 activity, resulting in abnormal protein accumulation and uncontrolled cell growth. GAS2DN competitively binds to Calpain-2, effectively displacing wild-type GAS2 and alleviating its inhibitory effect on Calpain-2 activity. This competitive interaction demonstrates specificity in inhibiting the growth of leukemia stem/progenitor cells 38, 49. GAS2DN features a conserved CH domain. It is plausible that specific peptide sequences within this domain might possess functionalities akin to GAS2DN, thus being termed GAS2 mimetic peptides. Targeting the GAS2-Calpain axis using these peptides could activate Calpain-2, thereby reducing abnormal protein accumulation and presenting a novel avenue for cancer therapy. Successful examples have demonstrated that it is feasible to deliver functional proteins or peptides into cells to achieve *in vitro* expansion of hematopoietic stem cells or inhibit tumor cell growth *in vivo* and *in vitro* 55-57. While recent years have seen significant progress in unraveling the functions of GAS2, there remains a notable gap in our understanding of its roles across various cancer types. Given the significance of GAS2 as a cytoskeleton-associated protein, it is imperative to explore its potential role in cancer progression through cytoskeletal regulation. The heterogeneity of cancer necessitates a nuanced investigation into how GAS2 functions within different tumor microenvironments and molecular contexts. Additionally, while the interaction between GAS2 and Calpain-2 has been well-documented, it is imperative to explore potential alternative mechanisms through which GAS2 may exert its effects on cancer occurrence and progression. Understanding these non-Calpain-2-dependent pathways could unveil novel therapeutic targets and broaden our strategies for cancer treatment. Therefore, further research efforts aimed at elucidating the multifaceted roles of GAS2 in different cancer types and uncovering alternative regulatory mechanisms are essential for advancing our comprehension of cancer biology and improving clinical outcomes.

## Supplementary Material

Supplementary figures and tables.

## Figures and Tables

**Table 1:** Summary of literature on the cytoskeleton, cell cycle, and apoptotic activities of GAS2.

Cytoskeleton	Cell cycle	Apoptosis
Gas2 is a component of the microfilament system 5.	Overexpression of Gas2 in Xenopus embryonic cells inhibited cell division 7	Gas2(∆276-314 and ∆236-314) alterations in the actin cytoskeleton and cellular morphology in NIH3T3 cells 11
Gas2 co-precipitated with microtubules 7	GAS2 suppressed the G1-to -S transition hepatocellular carcinoma SK-Hep1 cells 23, 30	GAS2 is up-regulated in BALB/c LT-2809 cells induced apoptosis when subjected to serum starvation 13
In COS-7 fibroblasts Gas2 C-terminus showed co- localization with the endogenous microtubule system 26	GAS2 promoted the G1-to-S transition of the cell cycle in T-ALL cell lines 33	Overexpression of Gas2 in U2OS cells and MEFs heightened apoptosis 12
		In MCF-7 cells GAS2's significance in inducing morphological changes in apoptotic cells 8

**Tabel 2:** Summary of literature on tumor promoting and tumor repressive activities of GAS2.

Tumor promoting activity	Tumor repressive activity
Cancers with GAS2 over-expression	Cancers with GAS2 down-Regulation
Acute myeloid leukemia 38	Breast cancer 22
Acute lymphoblastic leukemia 14, 38	Hepatocellular carcinoma 23, 30
Chronic myeloid leukemia 42, 43, 46, 47, 49	Prostate adenocarcinoma 24
Colorectal cancer 36, 37	
Signaling pathways involved	Signaling pathways involved
CXCR4 is the target of Calpain-2 14	p53 is the target of Calpain-2 9, 12, 30
β-catenin is the target of Calpain-2 46, 47	
Transcriptional regulation 49	
