# Supplementary material for: Insights into Structure and Function of Growth Arrest Specific 2 (GAS2)
Source: J Cancer. 2025 Jan 1;16(1):146–56. doi: 10.7150/jca.102893 (PMC11660135; doi:10.7150/jca.102893)
Supplement: Supplementary file 1 — Supplementary figures and tables. [file jcav16p0146s1.pdf]

Supplementary Table S1 Cancer abbreviations and corresponding full names in GEPIA2.

**abbreviation**	**full name**	**abbreviation**	**full name**	**abbreviation**	**full name**
ACC	adrenocortical carcinoma	BLCA	bladder urothelial carcinoma	BRCA	breast invasive carcinoma
CESC	cervical squamous cell carcinoma and endocervical adenocarcinoma	CHOL	cholangiocarcinoma	COAD	colon adenocarcinoma
DLBC	lymphoid neoplasm diffuse large B-cell lymphoma	ESCA	esophageal carcinoma	GBM	glioblastoma multiforme
HNSC	head and neck squamous cell carcinoma	KICH	kidney chromophobe	KIRC	kidney renal clear cell carcinoma
KIRP	kidney renal papillary cell carcinoma	LAML	acute myeloid leukemia	LGG	brain lower grade glioma
LIHC	liver hepatocellular carcinoma	LUAD	lung adenocarcinoma	LUSC	lung squamous cell carcinoma
OV	ovarian serous cystadenocarcinoma	PAAD	pancreatic adenocarcinoma	PCPG	pheochromocytoma and paraganglioma
PRAD	prostate adenocarcinoma	READ	rectum adenocarcinoma	SARC	sarcoma
SKCM	skin cutaneous melanoma	STAD	stomach adenocarcinoma	TGCT	testicular germ cell tumors
THCA	thyroid carcinoma	THYM	thymoma	UCEC	uterine corpus endometrial carcinoma
UCS	uterine carcinosarcoma				
